# West Nile virus keeps on moving up in Europe

**DOI:** 10.2807/1560-7917.ES.2020.25.46.2001938

**Published:** 2020-11-19

**Authors:** Tamás Bakonyi, Joana M Haussig

**Affiliations:** 1 European Centre for Disease Prevention and Control (ECDC), Stockholm, Sweden

**Keywords:** Europe, West Nile fever, West Nile virus, climate change, outbreaks, surveillance, travel, epidemiology

The 2020 arthropod vector season in Europe is approaching its end. Data from indicator-based surveillance on vector-borne diseases in 2020 will be reported to the European Centre for Disease Prevention and Control (ECDC) only in 2021, but we are already able to draw a preliminary picture from data obtained via event-based surveillance and weekly reporting of West Nile virus (WNV) infections.

In 2020, WNV caused remarkable outbreaks in certain areas in Europe, such as Spain and the Netherlands. However, the largest outbreak of human WNV infections in European Union/European Economic Area (EU/EEA) countries was recorded in 2018, when 11 countries reported 1,548 locally acquired mosquito-borne infections [1]. The number of WNV infections in 2018 exceeded the cumulative number of all reported infections between 2010 and 2017, and the highest number of newly affected areas (n = 45) was reported [2]. Even though in 2019, the number of reported locally acquired human WNV infections dropped by 73% compared with 2018, the total numbers were still the second highest ever recorded. Most countries reported numbers of infections similar to before 2018, while Greece continued to report a high number of infections [1].

Since the start of the 2020 transmission season on 1 June, and as at 12 November, EU/EEA countries have reported 315 human cases of WNV infection with known place of infection through the European Surveillance System (TESSy): Greece (n = 143), Spain (n = 77), Italy (n = 66), Germany (n = 13), Romania (n = 6), the Netherlands (n = 6), Hungary (n = 3) and Bulgaria (n = 1) [3] (Figure). It is noteworthy that some countries reporting very low numbers of infections in 2020, ranging from none to six, had previously detected higher numbers of human WNV infections (e.g. Austria, Hungary, Serbia, Romania). Additionally, in other endemic countries reporting a large number of cases (i.e. Greece and Italy), the proportion of the more severe manifestation of the infection, West Nile neuroinvasive disease (WNND), was higher than the average of the previous 5 years.

**Figure:**
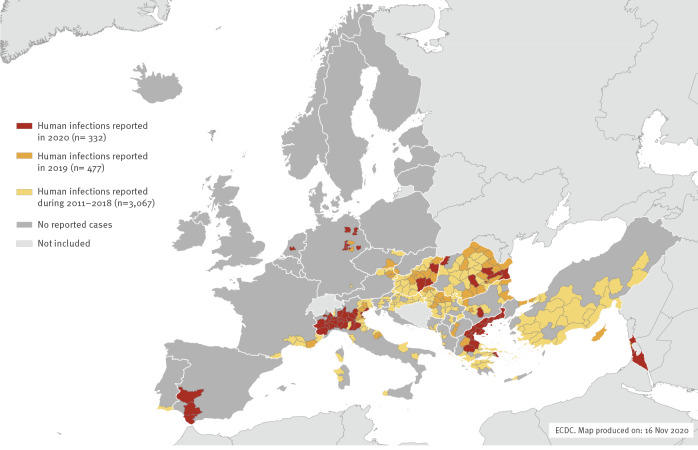
Distribution of locally acquired human West Nile virus infections by affected areas and transmission seasons, EU/EEA countries and EU neighbouring countries, 2011–2020^a^ (n = 3,876)

ECDC: European Centre for Disease Prevention and Control; EEA: European Economic Area; EU: European Union.

^a^ Data last updated on 16 November 2020.

Source: ECDC.

Within the last decade, the geographical spread of a genetic WNV lineage 2 strain has been observed in Central Europe and in the Mediterranean region [4]. In Germany, this EU-dominant strain was first detected in 2018 in resident birds and horses. The first five locally acquired vector-borne human cases were reported in 2019 in the country. In the current issue of *Eurosurveillance*, Pietsch et al. report an outbreak of nine locally acquired cases of West Nile fever (WNF) and WNND in Leipzig, Germany, in August and September 2020 [5]. The authors hypothesise endemic seasonal circulation of WNV lineage 2 in the city of Leipzig in 2020 and also in the coming years; therefore, they suggest population’s and healthcare workers’ WNV awareness should be further increased as well as the surveillance in animals.

WNV lineage 2 was also reported in bird and mosquito samples in the Netherlands, for the first time, at the end of August 2020 [6]. Hereafter, the first locally acquired human WNV infections were diagnosed, in the region of Utrecht, in September and October [3,7].

Since 2017, WNV lineage 2 has been spreading further west in the Mediterranean region, via the south of France, reaching Catalonia in north-eastern Spain, where it only caused sporadic cases in birds [8]. However, an unprecedented outbreak of WNV infections occurred in the southern Spanish provinces of Seville, Cádiz and Badajoz, between July and September 2020, comprising 77 infections diagnosed in humans and 137 documented outbreaks among equids [3]. A lineage 1 WNV strain was detected in both humans and animals, hence this outbreak has no epidemiological link to other concurrent WNV outbreaks in Europe [8]. Of note, Spain did not report locally acquired WNV infections in humans from 2017 to 2019.

Albeit numbers of human WNV infections in 2020 were lower than in previous years, the geographic expansion of WNV has continued in Europe. The environmental and ecological drivers of WNV are complex and not known in detail, yet. Nevertheless, ambient temperature is known as one important determinant through its effect on mosquito reproduction rates and the extrinsic virus period in mosquitoes [9]. According to the monthly climate bulletins of the Copernicus Climate Change Service [10], positive surface air anomalies were recorded in the southern regions of the Iberian Peninsula from July to August, as well as in north-west Europe from August to October. The temporospatial overlap with the WNV outbreaks might be a mere coincidence, but in the long term, the environmental conditions tend to become more favourable for WNV establishment and seasonal circulation in many European regions [9]. The European spread of another flavivirus, closely related to WNV, the Usutu virus (USUV), may be regarded as an example [11,12]. The first cases of USUV-associated wild bird mortality events were described in Italy in 1996 and in Austria in 2001. Within two decades, USUV has spread all over Europe, except for the Baltic countries and Scandinavia, and in 2020, USUV emerged in the United Kingdom [13]. Although the ecology and epizootiology of USUV and WNV differ in several points (e.g. higher genetic diversity of USUV in Europe indicates various, recent introductions from Africa), the two viruses share mosquito vectors and avian hosts; therefore, the environmental and ecological conditions suitable for USUV may indicate the same for WNV (e.g. vector competences [14]).

Most of the diagnosed human WNV infections in 2020 were reported from areas with virus activities already recorded in the previous years. This, together with genetic data, indicates overwintering and local circulation of the virus. Therefore, once established, the likelihood of maintenance and the risk of re-emergence of WNV infections in the affected European areas are high. The geographical spread after recent WNV lineage 2 introduction may be limited e.g. as it was seen in the eastern federal states of Germany, since 2018, but a massive geographical spread has been observed 4 to 5 years after its first emergence in Europe [4].

The annual fluctuations in WNV activity can be influenced by several factors. In the current issue of *Eurosurveillance*, Lourenço et al. have analysed West Nile virus epidemiology in Israel [15]. WNV infections caused by diverse strains have been diagnosed in the country in the past decade. In 2020, Israel reported 17 human cases as at 12 November. Authors adapted a suitability index to WNV and found that it confirmed the geotemporal estimation of transmission potential of WNV in the country. Several further studies have been and are investigating the ecological and environmental drivers of WNV [16,17]. Factors associated to national health systems, such as diagnostic awareness and vigilance, diagnostic capabilities and capacities, surveillance and reporting accuracies could also contribute to the annual variations of diagnosed and reported cases in a country. In 2020, the extraordinary and unprecedented burden on national health diagnostic systems caused by the coronavirus disease (COVID-19) pandemic might have had an influence on WNV surveillance in some countries.

The maintenance of national preparedness for seasonal WNV outbreaks in the forthcoming years is particularly important in countries where – even if sporadic – cases have been reported previously, as well as in countries where the ecological conditions are suitable for WNV emergence and establishment. The risk and public health impact of human WNV infections in the different European countries clearly and significantly varies. In certain countries, surveillance of human WNV infections might be challenging or of low priority. The natural cycle of WNV involves avian hosts and mosquito vectors, while – besides humans – equids are also frequent, incidental hosts of the virus. Therefore, integrated animal-human WNV surveillance with systematic response and control measures e.g. for blood donation safety, vector control, awareness and information campaigns, might allow a more efficient utilisation of national resources and capacities. Animal WNV infections should be diagnosed timely and EU/EEA countries are encouraged to report infections via the Animal Disease Notification System of the European Commission. These data are visualised together with the TESSy data on human cases in ECDC’s weekly updated maps on WNV infections in Europe [1]. The joint animal-human surveillance data can provide more accurate information on WNV activity within the transmission season than solely data from human infections. Joint efforts on WNV surveillance and control could be one of the good examples for a One Health approach towards zoonotic diseases.

## References

[1] European Centre for Disease Prevention and Control (ECDC). Surveillance atlas of infectious diseases. Stockholm: ECDC, [Accessed: 10 Nov 2020]. Available from: http://atlas.ecdc.europa.eu/public/index.aspx?Dataset=27&HealthTopic=60

[2] Young JJ Haussig JM Aberle SW Pervanidou D Riccardo F Sekulić N Epidemiology of human West Nile virus infections in the European Union and EU enlargement countries, 2010-2018. Euro Surveill. 2021; (Forthcoming). 10.2807/1560-7917.ES.2021.26.19.2001095 PMC8120798 33988124

[3] European Centre for Disease Prevention and Control (ECDC). Surveillance atlas of infectious diseases 2020. West Nile virus infection. Stockholm: ECDC. [Accessed: 10 Nov 2020]. Available from: https://atlas.ecdc.europa.eu/public/index.aspx?config=config-map-table&Header=None&Navigation=Embedded&Dataset=138&HealthTopic=60&GeoResolution=1&TimeResolution=Week&TimeSeriesRepresentation=T&FixDataset=1&FixHealthTopic=1

[4] Bakonyi T Ferenczi E Erdélyi K Kutasi O Csörgő T Seidel B Explosive spread of a neuroinvasive lineage 2 West Nile virus in Central Europe, 2008/2009. Vet Microbiol. 2013;165(1-2):61-70. 10.1016/j.vetmic.2013.03.005 23570864

[5] Pietsch C Michalski D Münch J Petros S Bergs S Trawinski H Autochthonous West Nile outbreak in Leipzig, Germany, August–September 2020. Euro Surveill. 2020;25(46):2001786 10.2807/1560-7917.ES.2020.25.46.2001786 PMC7678033 33213686

[6] Sikkema RS Schrama M van den Berg T Morren J Munger E Krol L Detection of West Nile virus in a common whitethroat (Curruca communis) and Culex mosquitoes in the Netherlands, 2020. Euro Surveill. 2020;25(40). 10.2807/1560-7917.ES.2020.25.40.2001704 33034280 PMC7545818

[7] Vlaskamp DRM Thijsen S Reimerink J Hilkens P Bouvy WH Bantjes SE First autochthonous West Nile virus infections in the Netherlands; from one to six cases, July to August 2020. Euro Surveill. 2020;25(46):20-01904. 10.2807/1560-7917.ES.2020.25.46.20-01904 PMC7678035 33213687

[8] Ministry of Health Spain. Centro de Coordinación de Alertas y Emergencias Sanitarias. Evaluación rápida de riesgo: Meningoencefalitis por el virus del Nilo occidental en España (1ª actualización). [Rapid risk assessment: Meningoencephalitis caused by West Nile virus in Spain (1^st^ update)]. Madrid: Ministry of Health; 9 Oct 2020. Spanish. Available from: https://www.mscbs.gob.es/profesionales/saludPublica/ccayes/alertasActual/docs/20201009_ERR_Nilo_Occidental.pdf

[9] Paz S Semenza JC Environmental drivers of West Nile fever epidemiology in Europe and Western Asia--a review. Int J Environ Res Public Health. 2013;10(8):3543-62. 10.3390/ijerph10083543 23939389 PMC3774453

[10] Copernicus Climate Change Service. (C3S). Climate bulletins. [Accessed: 10 Nov 2020]. Available from: https://climate.copernicus.eu/monthly-climate-bulletins

[11] Clé M Beck C Salinas S Lecollinet S Gutierrez S Van de Perre P Usutu virus: A new threat? Epidemiol Infect. 2019;147:e232. 10.1017/S0950268819001213 31364580 PMC6625183

[12] Vilibic-Cavlek T Petrovic T Savic V Barbic L Tabain I Stevanovic V Epidemiology of Usutu Virus: The European Scenario. Pathogens. 2020;9(9):E699. 10.3390/pathogens9090699 32858963 PMC7560012

[13] Folly AJ Lawson B Lean FZ McCracken F Spiro S John SK Detection of Usutu virus infection in wild birds in the United Kingdom, 2020. Euro Surveill. 2020;25(41). 10.2807/1560-7917.ES.2020.25.41.2001732 33063656 PMC7565854

[14] Martinet JP Ferté H Failloux AB Schaffner F Depaquit J Mosquitoes of North-Western Europe as Potential Vectors of Arboviruses: A Review. Viruses. 2019;11(11):E1059. 10.3390/v11111059 31739553 PMC6893686

[15] Lourenço J Thompson RN Thézé J Obolski U Characterising West Nile virus epidemiology in Israel using a transmission suitability index. Euro Surveill. 2020;25(46):1900629 10.2807/1560-7917.ES.2020.25.46.1900629 PMC7678037 33213688

[16] Gangoso L Aragonés D Martínez-de la Puente J Lucientes J Delacour-Estrella S Estrada Peña R Determinants of the current and future distribution of the West Nile virus mosquito vector Culex pipiens in Spain. Environ Res. 2020;188:109837. 10.1016/j.envres.2020.109837 32798954

[17] Rosalie PA Bisesi M Novel Indices of Meterological Drivers of West Nile Virus in Ohio Culex Species Mosquitoes From 2002-2006. J Environ Health. 2017;79(8):16-22. 29148653

